# Coronavirus disease 2019 vaccine hesitancy among children’s hospital staff: A single-center survey

**DOI:** 10.1017/ice.2021.58

**Published:** 2021-02-09

**Authors:** Larry K. Kociolek, Jenny Elhadary, Ravi Jhaveri, Ami B. Patel, Brian Stahulak, Jenifer Cartland

**Affiliations:** 1 Ann & Robert H. Lurie Children’s Hospital of Chicago, Chicago, Illinois; 2 Northwestern University Feinberg School of Medicine, Chicago, Illinois

The Pfizer-BioNTech COVID-19 (BNT162b2) vaccine was authorized for emergency use on December 11, 2020,^1^ after demonstrating excellent efficacy and safety in a large phase 3 clinical trial in adults and adolescents.^2^ The Advisory Committee on Immunization Practices through the Centers for Disease Control and Prevention then recommended its use,^1^ prompting delivery to US hospitals for healthcare worker vaccination. To help guide our children’s hospital workforce vaccine advocacy efforts, we designed a survey assessing frequency of vaccine hesitancy, characteristics of those reporting vaccine hesitancy, specific concerns, and communication preferences.

## Methods

This survey was performed at the Ann & Robert H. Lurie Children’s Hospital of Chicago, a 360-bed academic free-standing children’s hospital with multiple satellite outpatient and surgical centers. On December 21, 2020, the first day of administration of the Pfizer-BioNTech COVID-19 vaccine to our workforce, a 17-question electronic survey (Supplemental Material online) was sent to all individuals with an active Lurie Children’s email address. This survey included all clinical (eg, attending physicians, housekeeping staff, advanced practice staff, and ancillary healthcare workers), and nonclinical (eg, administrative, support, and research) staff. After 3 e-mail reminders were sent, the survey was closed on January 13, 2021. The survey was anonymous, and all questions were optional. During the 2 weeks before the survey, vaccine information was communicated to staff through a virtual town hall, and answers to frequently asked questions were emailed to all staff. Statistical analyses were performed using Stata/IC version 16.0 software (StataCorp, College Station, TX). Descriptive statistics were measured, prevalence ratios were calculated, and proportions were compared using the χ2 test. Two-sided *P* values < .05 were considered statistically significant. Variables that were statistically significant on bivariate analysis were analyzed by multivariate logistic regression.

This study was exempt from institutional review board review as a quality improvement initiative using anonymous data.

## Results

The survey was sent to 7,012 individuals, and 4,448 responded (response rate, 63.4%). Most reported that they will definitely receive the vaccine (n = 2,559, 59.8%), and 368 (8.6%) had already received the vaccine. Vaccine hesitancy, defined as those reporting that they definitely will not (n = 193), probably will not (n = 185), or have not yet decided (n = 432) whether they will receive the COVID-19 vaccine, was reported in 810 of 4,277 respondents (18.9%). Table 1 identifies demographics, employment characteristics, and COVID-19 perceptions associated with vaccine hesitancy. Among those who reported vaccine hesitancy, Table S1 (online) lists the prevalence of specific concerns, and Tables S2 and S3 (online) list the preferred COVID-19 vaccine educational resources and information dissemination methods.

**Table 1.** Demographics, Employment Characteristics, and COVID-19 Perceptions Associated With Vaccine Hesitancy

Characteristic	Vaccine Hesitant, No. (%)	Not Vaccine Hesitant, No. (%)	Prevalence Ratio (95% CI)
**Sex**			
Female (n=3,377)	668 (19.8)	2,709 (80.2)	1.7 (1.4–2.2)*
Male (n=759)	86 (11.3)	673 (88.7)	
**Race**			
Black (n=411)	207 (50.4)	204 (49.6)	3.2 (2.9–3.6)*
Not black (n=3,866)	603 (15.6)	3,263 (84.4)	
**Ethnicity**			
Hispanic/Latinx (n=612)	183 (29.9)	429 (70.1)	1.7 (1.5–2.0)*
Not Hispanic/Latinx (n=3,665)	627 (17.1)	3,038 (82.9)	
**Age, y**			
≤40 (n=2,312)	423 (18.3)	1,889 (81.7)	0.99 (0.87–1.1)
≥41 (n=1,855)	344 (18.5)	1,511 (81.5)	
**History of COVID-19**			
Confirmed or suspected history of COVID-19 (n=602)	181 (30.1)	421 (69.9)	1.8 (1.5–2.0)*
No history of COVID-19 (n=3,667)	625 (17.0)	3,042 (83.0)	
**Personal concern for COVID-19 risk**			
Not concerned about severe COVID-19 (n=2,514)	489 (19.5)	2,025 (80.5)	1.7 (1.4–2.0)*
Concerned about severe COVID-19 (n=1,148)	133 (11.6)	1,015 (88.4)	
**High-risk medical conditions**			
Yes (n=459)	214 (46.6)	245 (53.4)	3.0 (2.7–3.4)*
No or unsure (n=3,795)	585 (15.4)	3,210 (84.6)	
**Medical center role**			
Nonclinical (n=1,615)	463 (28.7)	1,152 (71.3)	2.4 (2.1–2.8)*
Clinical (n=2,568)	305 (11.9)	2,263 (88.1)	
**Employment type**			
Hourly employee (n=1,879)	519 (27.6)	1,350 (72.4)	2.4 (2.1–2.8)*
Salaried employee (n=2,317)	264 (11.4)	2,053 (88.6)	

Note. CI, confidence interval.

* *P* < .01 on both bivariate and multivariate analysis.

## Discussion

We identified COVID-19 vaccine hesitancy among nearly 20% of our children’s hospital work force. Vaccine hesitancy was more prevalent among members of our work force who identify as female, black, and/or Hispanic/Latinx. These race- and ethnicity-related associations have been described for COVID-19 vaccination^3^ and are particularly concerning given the disproportionate impact of COVID-19 incidence and severity in black and Hispanic/Latinx populations in the United States.^4^ Not surprisingly, vaccine hesitancy was associated with less concern about personal risk of severe COVID-19. Unexpectedly, vaccine hesitancy was 3 times more prevalent among individuals who identify themselves as having high-risk medical conditions, further highlighting the need for vaccine advocacy efforts among vulnerable patient populations. We identified specific COVID-19 concerns, especially concerns about vaccine safety related to novelty and speed of the clinical development process. Those expressing vaccine hesitancy identified their own medical doctor and national and local experts as trustworthy sources who can influence their vaccine decisions. Notably, social media was not valued as a tool for vaccine information, which is reassuring given the role of social media in disseminating vaccine misinformation.^5^

These data have equipped our COVID-19 response team with knowledge to develop targeted vaccine education and advocacy strategies. With these data, we have planned the following 5-part program for our work force: (1) small group discussions with members of our environmental services, security, and food services department employees led by infectious diseases experts and physician leaders who identify as black and/or Hispanic/Latinx; (2) website link with answers to frequently asked questions that is accessible by smartphone; (3) vaccine information brochures in Spanish and English; (4) regular e-mail updates and virtual town hall meetings with question and answer sessions; and (5) videos of hospital clinical and administrative leaders receiving and discussing their decision to receive the vaccine. We plan additional advocacy and education efforts for the community.

Although this survey was performed after vaccine safety and efficacy data were available and initiation of hospital vaccine education efforts, the frequency of vaccine hesitancy was similar to the 22% vaccine hesitancy prevalence among nearly 2,000 non–healthcare workers in the United States who were surveyed prior to authorization of COVID-19 vaccines in the United States.^3^ Although the survey was limited to a single pediatric center, this frequency of hesitancy may suggest that our data may be generalizable beyond our center.

This study has several limitations. Although the response rate was robust, we failed to receive responses from approximately one-third of our workforce. Because this survey was anonymous, we were unable to determine the characteristics of nonrespondents compared to respondents. The survey was completed immediately after the authorization of COVID-19 vaccine in the United States. It is unclear whether ongoing vaccine experience and uptake have impacted vaccine hesitancy since that time.

In summary, through an electronic survey of our children’s hospital workforce, we identified characteristics associated with vaccine hesitancy and identified specific concerns and communication preferences of our vaccine hesitant staff. These data have guided development of targeted vaccine education and advocacy strategies to improve the health of our workforce and safety of our healthcare environment.

## Supplementary material

For supplementary material accompanying this paper visit https://doi.org/10.1017/ice.2021.58. click here to view supplementary material
