# Supplementary material for: Coronavirus disease 2019 vaccine hesitancy among children’s hospital staff: A single-center survey
Source: Infect Control Hosp Epidemiol. 2021 Feb 9:1–3. doi: 10.1017/ice.2021.58 (PMC7925985; doi:10.1017/ice.2021.58)
Supplement: Supplementary file 1 [file S0899823X21000581sup001.docx]

**SUPPLEMENTARY TABLES**

**Table S1: COVID-19-related concerns among individuals reporting vaccine hesitancy**

| **Concern** | **n (%)** |
| --- | --- |
| The research has gone too fast to know if there are long term side effects | 618 (76.3) |
| It might not be safe for me | 411 (50.7) |
| The mRNA method is too new | 387 (47.8) |
| The process of creating the vaccine was too political, and I worry the FDA and CDC might not be telling the truth as a result | 355 (43.8) |
| The vaccine has not been tested on people like me (my age, race or ethnicity, or people with my medical history) | 249 (30.7) |
| The vaccine might not prevent spread of the virus | 223 (27.5) |
| Immunity might not last long enough | 189 (23.3) |
| It might not work for me | 94 (11.6) |
| I have had COVID-19 and/or a lab test that detected COVID-19 antibodies, so I'm not sure why I would need the vaccine | 52 (6.4) |

**Table S2: Most important resources for COVID-19 vaccine information among individuals reporting vaccine hesitancy**

| **Resource** | **n (%)** |
| --- | --- |
| Self-guided research on the vaccine | 377 (46.5) |
| Primary care provider | 267 (33) |
| The federal government and agencies | 233 (28.8) |
| Hospital leadership and vaccine experts | 183 (22.6) |
| Coworkers or friends who get the vaccine | 126 (15.6) |
| Local public health officials | 92 (11.4) |
| Hospital experts | 81 (10) |
| Newspapers and news programs on television or internet | 43 (5.3) |
| Social media | 16 (2) |

**Table S3: Preferred communication method for COVID-19 vaccine information from hospital leaders among individuals reporting vaccine hesitancy**

| **Communication Method** | **n (%)** |
| --- | --- |
| Website for self-learning | 380 (46.9) |
| Email updates | 297 (36.7) |
| Video updates from hospital leaders and vaccine experts | 281 (34.7) |
| Town hall meetings with questions and answers | 242 (29.9) |
| Small group discussions with questions and answers | 103 (12.7) |
| Updates from direct supervisor | 85 (10.5) |
| Podcasts from hospital experts | 63 (7.8) |
| Grand rounds with questions and answers | 47 (5.8) |
